# Down-regulation of miR-302b, an ESC-specific microRNA, in Gastric Adenocarcinoma

**Published:** 2011-12-22

**Authors:** Mitra Khalili, Majid Sadeghizadeh, Kamran Ghorbanian, Reza Malekzadeh, Mohammad Vasei, Seyed Javad Mowla

**Affiliations:** 1. Department of Molecular Genetics, Faculty of Biological Sciences, Tarbiat Modares University, Tehran, Iran; 2. Hematology-Oncology and Stem Cell Research Center, Shariati Hospital, Tehran University of Medical Sciences, Tehran, Iran; 3. Digestive Disease Research Center, Shariati Hospital, Tehran University of Medical Sciences, Tehran, Iran; 4. Pathology Laboratory, Shariati Hospital, Tehran University of Medical Sciences, Tehran, Iran

**Keywords:** Cancer Stem Cells, MicroRNA, MiR-302b, Gastric Cancer

## Abstract

**Objective:**

microRNAs (miRNAs) are a new class of non-coding RNAs involved in regulating various biological processes including proliferation, differentiation, and apoptosis, among others. Alterations in miRNA expression are reported in several human cancers, which suggests their potential roles in tumor initiation and progression. Members of the miR-302 cluster are highly expressed in embryonic stem cells (ESC), where they regulate cell self-renewal and pluripotency. Based on the cancer stem cell (CSC) hypothesis, mis-expression of such genes might contribute to tumorigenicity. This study aims to find a potential link between the expression level of human/homo sapiens miR-302b (has-miR- 302b) and tumor/grade state of gastric tissues.

**Materials and Methods:**

A matched based case-control study was conducted that included tumor and matched marginal non-tumor surgical specimens from 34 patients diagnosed with gastric adenocarcinoma. Randomly selected samples were obtained from the Iran National Tumor Bank. cDNA synthesis was carried out on total RNA, by using the miRCURY LNA^TM^ Universal RT microRNA PCR Kit. Real-time reverse transcriptionpolymerase chain reaction (RT-PCR) assays were performed with specific LNA^TM^ primers and SYBR Green master mix. The human embryonic carcinoma cell line, NTERA2 (NT2) and a human gastric adenocarcinoma cell line, AGS, were used to optimize the PCR reactions. A comparative evaluation of miR-302b expression in tumor and non-tumor gastric samples was performed by either paired t test or Wilcoxon non-parametric test. The ability of miR-302b to discriminate tumor from non-tumor gastric samples was evaluated using the area under the receiver operating characteristic (ROC) curve.

**Results:**

According to our data, miR-302b expression (normalized to that of the U6 snRNA housekeeping gene) in the pluripotent cell line NT2 was more than 500 times greater than that of the AGS cell line. The level of expression was even lower in tumor and non-tumor gastric tissue samples. The data further revealed a down-regulation of miR-302b in gastric tumor samples (p=0.001), particularly in high-grade adenocarcinoma (p=0.009). However, ROC analysis data demonstrated a low sensitivity and specificity of miR-302b expression to discriminate between the tumor and non-tumor state of the samples (AUC=0.63).

**Conclusion:**

Despite the upregulation of some hESC-specific genes in tumors, our data revealed a down-regulation of miR-302b in high-grade tumors. This data suggested a potential tumor-suppressor role for miR-302b in tumorigenesis of gastric tissue.

## introduction

Gastric cancer is the fourth most common malignancy and the second cause of cancer-related death in the world (1, 2). Multiple genetic alterations are involved in the pathogenesis of gastric cancer, including the elevated expression/activation of oncogenes and/or down-regulation/inactivation of tumor suppressor genes. Recently, re-expression of a number of stem cell-specific genes has been reported in some cancers. Based on the cancer stem cell (CSC) hypothesis, CSCs have originated either from normal adult stem cells that acquired deregulated self-renewal control or from transformed somatic progenitor/differentiated cells reprogrammed into a stemness state (3-5).

MicroRNAs (miRNAs), are a new class of endogenously made non-coding RNAs with a small size of ~22-nt. They mostly bind to the 3'-untranslated region (3'-UTR) of their target mRNAs, inhibiting their translation or decreasing their stability. It is estimated that more than a third of all human genes are regulated by miRNAs (6). miRNAs are involved in regulating various cellular processes including cell cycle, proliferation, and apoptosis (7-9). The fact that the impairments of such cellular processes are vital ingredients of tumorigenicity has led to the speculation that miRNAs may have a potential role in tumor initiation and progression (10). Depending on their specific target genes, miRNAs can function as either tumor suppressors or oncogenes (11). miRNA deregulation has been reported in several tumors including colon (12), prostate (13), lung (14), breast (15), liver (16), pancreas (17), and bladder (18).

Members of the miR-302 cluster (miR-302a, miR-302b, miR-302c, miR-302d, and miR-367) are the most abundant miRNAs in human embryonic stem cells (hESCs). Functionally, they regulate self-renewal and pluripotency processes, and therefore represent a master regulatory role in the stemness maintenance of ESCs (19). Interestingly, the ESC-specific transcription factors OCT4, SOX2, Nanog and Rex-1 have binding sites on the miR-302 promoter, thus regulating its expression (20, 21).

Despite the current diagnostic and therapeutic advancements, gastric cancer remains the second reason for cancer-related death in the world; and little improvement in patient survival has been so far achieved (22). Many different molecular alterations in protein coding and/or non-coding genes have been shown to be involved in gastric carcinogenesis. Unveiling such molecular pathways would lead to the development of new methods for better diagnosis, prognosis, and treatment of this cancer.

The present study has taken into consideration: 1. the CSC hypothesis, 2. the fact that the expressions of some miRNAs are altered in cancers, and 3.the high stability and ease of detection of miRNAs in clinical samples as a potential new class of tumor markers. Therefore, we have employed a real-time reverse transcription–polymerase chain reaction (RT-PCR) approach to compare the expression level of miR-302b in a series of tumor and non-tumor gastric specimens. The obtained data have a potential for better identification and classification of gastric cancer.

## Materials and Methods

### Cell lines and cell culture

We obtained the human gastric adenocarcinoma cell line AGS from the National Cell Bank of Iran (Pasteur Institute of Iran, Tehran). AGS was cultured in RPMI-1640 (Gibco, USA) medium, supplemented with penicillin (100 U/ml), streptomycin (100 µg/ml), and 10% fetal bovine serum (FBS), at 37℃ in a humidified atmosphere of 5% CO_2_. The human embryonic carcinoma cell line NTERA2 (NT2; kindly provided by Dr Peter Andrews at Sheffield University) was cultured in Dulbecco's modified eagle medium (DMEM) with a high concentration of glucose, supplemented with 10% FBS, at 37℃ in a humidified atmosphere of 5% CO_2_.

### Patients and clinical samples

We performed a matched case-control study in which 34 randomly selected pairs of gastric samples, including gastric adenocarcinoma and their matched non-tumor tissue samples, were obtained from the Iran National Tumor Bank. The sample size was calculated based on the assumption of 1 ΔCT difference for miR-302 expression between tumor and non-tumor gastric samples, with the consideration of a type I error of 0.05 and type II error of 0.20. The samples had been immediately snap-frozen in liquid nitrogen and stored at -70ºC until RNA extraction. For each patient, we collected clinico-pathological information that included gender, age, and tumor stage based on the TNM system, where: T is the extent of the primary tumor, N, the amount of regional lymph node involvement and M, distant metastasis (Table 1).

**Table 1:** Clinico-pathological characteristics of patients with gastric cancer


Samples characteristics			
Gender	Female	14	41%
	Male	20	59%
Age (year)	>55	26	76.5%
	≤55	8	23.5%
	Median	63 ± 9	
Differentiation	Low Grade=I-II	16	47%
	High Grade=III	18	53%
Lymph node metastasis	N0	7	20%
	N1	18	53%
	N2	7	20.5%
	N3	2	6%
Invasion depth	T2	6	17.60%
	T3	27	79.4%
	T4	1	2.9%
Distance metastasis	M0	23	67.5%
	M1	8	23.5%
	Unknown	3	9%


A total of 34 pairs of gastric samples, including gastric adenocarcinoma and their matched non-tumor tissue samples, were collected. *Tumor staging was determined by the TNM system. The system is based on the extent of the tumor (T), the extent of spread to the lymph nodes (N), and the presence of distant metastasis (M). A number is added to each letter to indicate the size or extent of the primary tumor and the extent of cancer spread.

The experimental procedures were approved by the Ethical Committees of Imam Khomeini Hospital and Tarbiat Modares University. Representative formalin-fixed paraffin-embedded (FFPE) sections for each sample were stained with hematoxylin and eosin (H&E) for verification of tumor versus nontumor sample states, as well as their malignancy grades.

### **RNA** extraction and real-time **RT-PCR** assay

Total RNA was isolated from the homogenized tissue specimens, using TRIzol® reagent (Invitrogen, USA), according to the manufacturer's instruction. RNA yield and A260/280 ratio were determined by a NanoDropND-100 spectrometer (Thermo Fisher Scientific, USA). RNase-free DNase (Takara, Japan) treatment of total RNA was performed to eliminate any potential contamination with genomic DNA. cDNA synthesis was carried out on 200 ng of total RNA, by using the miRCURY LNA^TM^ Universal RT microRNA PCR Kit (Exiqon, Denmark). The tube was incubated for 60 minutes at 42℃, followed by heat-inactivation of the reverse transcriptase enzyme for 5 minutes at 95℃. Afterward, real-time RT-PCR was performed, using 1µL of cDNA product, mir-302b LNA^TM^ primers (Exiqon, Denmark), and SYBR Green master mix (Exiqon, Denmark). The U6 sn- RNA gene was used as an internal control. PCR reactions were conducted at 95℃ for 10 minutes, followed by 40 cycles of 95℃ for 10 seconds, and 60℃ for 1 minute in an ABI 7500 real-time quantitative PCR system (Applied Biosystems, USA). All real-time PCR reactions were performed in duplicates. To minimize data variation in separate runs, paired tumor and non-tumor samples from the same patient were examined on the same runs. To ensure that the RNA samples were not contaminated with genomic DNA, we included a no reverse transcriptase control (no RT) during each run of real-time RT-PCR. Furthermore, to check the accuracy of amplifications, we included a negative control in each run by eliminating the cDNA sample in the tube.

To determine the reaction efficiencies for each primer pair, we used LinRegPCR (12.x) software (AMC, Amsterdam, http://LinRegPCR.nl), a program for analyzing real-time PCR data. LinReg PCR software measures the efficiency of PCR along every cycle of one run. We also plotted standard curves by using serial dilutions of NT2 cDNA, as a positive control for miR-302b expression, and by using ABI 7500 software (Applied Biosystems, USA).

### Statistical analyses

Real-time RT-PCR data was adjusted based on the exact PCR efficiency. For calculation of miR-302b expression fold change, the expression level of miR-302b in each sample was normalized to that of U6 snRNA, as an internal control. Then, miR-302 expression in tumor samples was normalized to the matched non-tumor samples (2^‸‑ΔΔCT^). For evaluation of miR-302b expression in tumor and non-tumor groups, in each sample the expression level of miR-302b was normalized to that of U6 snRNA, then the level of expression of each sample was calibrated to that of the least expressed sample. For statistical analysis, initially the Kolmogorov–Smirnov normality test (KS-test) was used to examine the normal distribution of the samples. Then, statistical differences between tumor (high and low grades) and matched non-tumor gastric samples were determined by paired t-test (if samples passed the KS-test) or Wilcoxon non-parametric test (for samples that did not pass the KS-test). The difference between the grades of gastric samples and miR-302b expression fold change was determined by the Mann-Whitney non-parametric test. All tests were performed as two-tailed and a p value of <0.05 was considered statistically significant.

Receiver operating characteristics (ROC) curve was plotted to determine how well the expression level of miR-302b discriminated between tumor and non-tumor gastric samples.

Real-time RT-PCR data was normalized and analyzed with GenEX software (MultiD Analyses AB, Goteborg, Sweden), and Statistical Program for Social Sciences (SPSS) software version 17 (SPSS Inc., Chicago, IL, USA).

## Results

### Optimizing the amplification of miR-302b in NT2 and AGS cell lines

**Fig 1:**
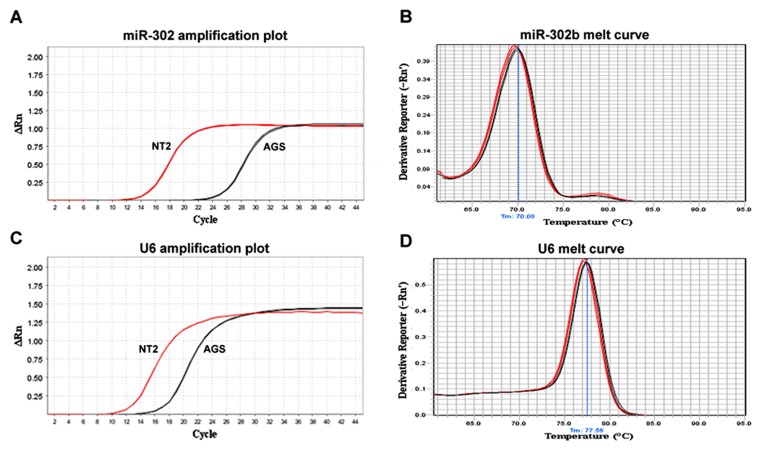
miR-302b and U6 snRNA expression in AGS gastric cancer and NT2 human embryonic cancer cell lines. A and C; Representative amplification plots of mir-302b (A) and U6 snRNA (internal control, C) for NT2 and AGS cell lines. Note that the expression of miR-302b is significantly higher (~500x) in NT2 cells compared to that of AGS cells. B and D; The corresponding melt curves of miR-302b and U6 miR-302b (B) and U6 snRNA (D) the PCR products confirmed the specificity of the primers to amplify exact targets in both cell lines.

Before we examined miR-302b expression in gastric tissues, the real-time RT-PCR procedure was optimized on NT2 as a positive control. We also used the human AGS cell line to compare the level of miR-302b expression in a gastric cancer cell line compared to that of a pluripotent cell line. As shown in Figure 1A, the relative expression of miR-302b (normalized to that of U6 snRNA as an internal control; figure 1C) in NT2 cells was ~500 times higher than that of the AGS cell line. The PCR products in both cell lines produced identical melt curves for both miR-302b and U6 snRNA (Fig 1B ,D), which confirmed that the employed LNA primers had high specificity for the detection of miR-302b expression, with no cross-reactivity to other miRNAs. There were no PCR products in the negative and “no RT” controls.

### MiR-302b expression in gastric tumors and their matched non-tumor tissues

**Fig 2:**
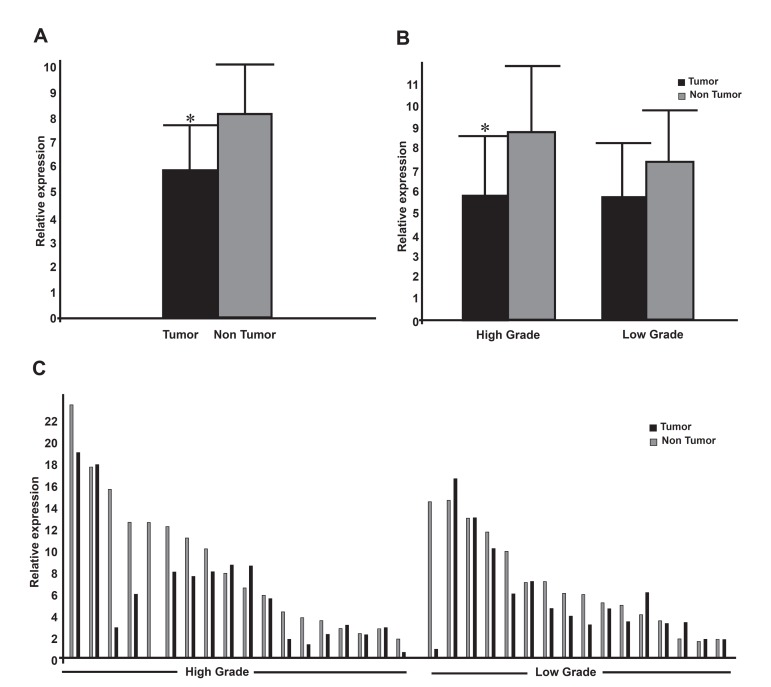
MiR-302b expression in tumor vs. non-tumor gastric samples. In each sample, the expression level of miR-302b is normalized to that of U6 snRNA, as an internal control. The level of expression of each sample is also calibrated to that of the least expressed sample. A. Histograms show the mean values of miR-302b's relative expression in tumor and non-tumor samples, with confidence interval as an error bar. Note the expression of miR-302b is significantly downregulated in tumor samples compared to their non-tumor counterparts (p=0.001). B. Comparative expression of miR-302b in different grades of gastric samples. Note that only the relative expression of miR-302b in tumors with high grad of malignacy is significantly down-regulated (p=0.009). C. The relative expression of miR-302b in individual samples, distributed in high and low grade groups.

Next, we determined the relative expression of miR-302b in 34 paired tumor/non-tumor gastric tissue surgical specimens. The clinicopathological characteristics of the patients are summarized in table 1. The expression level of miR-302b was low in both tumor and non-tumor samples. Thus, the optimal concentration of total RNA for cDNA synthesis was empirically determined in the range of RNA concentrations suggested by the manufacturer. It was determined that a start concentration of 200 ng of total RNA

and 1µl of the related cDNA generated detectable CT values for miR-302b (35.5 ± 2) and the internal control U6 snRNA (19.5 ± 4). There was no amplification in the negative and no-RT control samples (CT was undetermined). In contrast, NT2 cells as the positive control, had a high expression of miR-302b with a CT of 16 ± 2 (Fig 1A). As shown in figure 2A, the expression level of miR-302b was down-regulated in tumor samples compared to their non-tumor counterparts obtained from the same patients, by a p value of 0.001 (non-parametric Wilcoxon test). Distributing the data in different grades of malignancy revealed that the relative expression of miR-302b mostly declined in tumors that had a high grade of malignancy by a p value of 0.009 (paired ttest), while the observed down-regulation in the low-grade samples was not statistically significant (p=0.10, non-parametric Wilcoxon test; Fig 2B). The relative expression of miR-302b in individual samples, distributed in high and low grade groups, is presented in figure 2C. The obtained data failed to show any significant difference between the relative expression of miR- 302b in high and low grade gastric tumors, when adjusted to the expression of their matched nontumor samples (2^‸‑ΔΔCT^; p=0.33, non-parametric Mann-Whitney).

### Association of miR-302b expression with patients' clinico-pathological data

**Fig 3:**
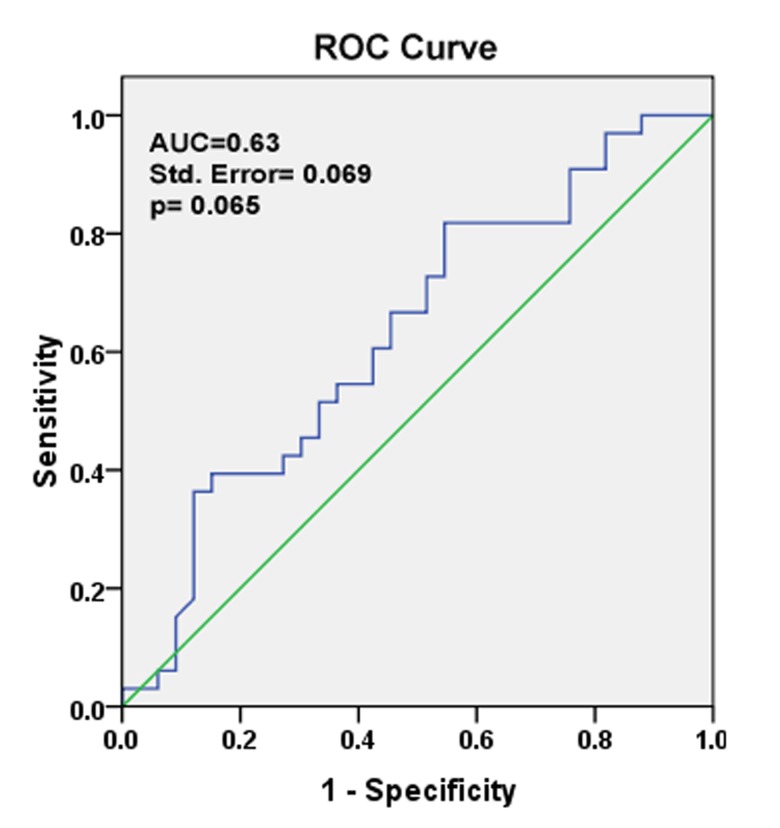
ROC curve analysis for testing the sensitivity and specificity of miR-302b expression to discriminate between tumor and non-tumor states of the samples. Area under curve (AUC=0.63) shows that data from miR-302b expression does not have high ability to correctly identify and distinguish tumor versus non-tumor groups of gastric samples.

We used ROC analysis to evaluate the suitability of miR-302b expression to discriminate between the tumor and non-tumor state of the samples. Total area under the curve (AUC) for miR-302b was 63% (p=0.065; Fig 3). Larger AUC value means better overall performance of the medical test to correctly distinguish tumor and non-tumor samples. Thus, it seemed that miR-302b, as a tumor marker, did not have adequate sensitivity and specificity to discriminate between tumor and nontumor gastric samples.

## Discussion

Recently we reported a re-expression of OCT4, a well-known embryonic stem cell (ESC), in both bladder (23) and gastric cancers (24). OCT4, also known as OCT3 and POU5F1, is a master regulator of stemness state, self-renewal, and pluripotency in stem cells (25-27). Its re-expression in tumor samples can be regarded as evidence to support the CSC hypothesis in these tumors. These and other reports on expression of OCT4 in cancer cell lines and tissues (23, 28) have appeared to be highly controversial. Several other reports claim that OCT4 is exclusively expressed in ESCs with no expression in adult stem cells, cancer cell lines, and tumor tissues (29, 30). A potential source of controversy is probably generated by the presence of several expressed OCT4 pseudogenes (31, 32), or the failure of techniques to discriminate between the expressions of different variants of OCT4. Therefore, finding a better hESC-specific marker would generate a more valid and reproducible mean to evaluate the pluripotency state of stem and CSC in labs and clinics.

It has been already shown that the expression of miRNAs is cell- and tissue-specific (33), of which some are exclusively expressed in ESCs (34). The cluster of miR-302 is the most abundantly expressed miRNA in undifferentiated ESCs, and its expression is sharply turned off upon the induction of differentiation (20). The miR-302 promoter has binding sites for the main ESC-specific transcription factors, i.e. OCT4, Nanog, Sox2, and Rex1 (20, 21). The members of the cluster regulate the cell cycle in ESCs, promote self-renewal and pluripotency of the cells, and hence participate in the maintenance of ESCs (19). However, their potential role in induction of pluripotency pathways in somatic cells for generation of CSCs and initiation of tumorigenicity is still ambiguous. Thus, in this study we have evaluated a potential alteration in the expression of miR-302b, the main regulatory miRNA in reprogramming and pluripotency pathways (19, 35) in gastric tumor samples.

For detection of miR-302b, we used Locked-Nucleotide Acid (LNA) primers, which provided high affinity and excellent discriminative power for the specific amplification of target miRNAs (36).

As expected, we detected a high expression level of miR-302b in the NT2 cell line, the positive control in our study. Interestingly, we also detected miR-302b expression in the AGS cell line, albeit at a level 500 times less than in NT2 cells. The low expression of the miR-302b in AGS cells could be due to the restricted expression of miRNA in a rare subpopulation of the cells, such as CSCs. A similar low and restricted expression of miR-302b in some glioma cell lines has been reported (37). Our data also revealed a lower expression of miR- 302b in tumor and non-tumor gastric samples. A comparison of the pattern of miR-302b expression in NT2, AGS, and gastric tumor/non-tumor tissue samples to the pattern of OCT4 expression in the same samples (24) has suggested that miR-302b could be considered a better marker of pluripotency. In NT2 and other pluripotent cells, high expression of miR-302 coincides with high expression of OCT4. There is a positive correlation between miR-302 expression and induced pluripotency (ips) genes, including OCT4 variants, in gastric adenocarcinoma. The expression pattern of miR-302 and ips genes greatly varies among the diffuse versus intestinal, subgroups of tumors (Unpublished data).

While we expected an elevated expression of miR-302b in tumor samples compared to their nontumor counterparts, a significant down-regulation of the gene interestingly appeared in tumor samples. However, this finding was in agreement with a recent report by Lin et al. (38) who have demonstrated tumor suppressive activity for miR-302. They claimed that miR-302 is a human tumor suppressor capable of attenuating rapid cell growth, causing tumor cell apoptosis, and inhibiting tumor cell invasion and metastasis.

We also expected to see a higher expression of miR-302b at higher grades of malignancy, where the cells are mostly in a poorly differentiated state. In contrast, while the expression of miR-302b was down-regulated in all grades of malignancies, the most significant decrease was observed in highly malignant tumors. The later finding raised a hypothesis that tumor progression might require the silencing or down-regulation of miR-302b in its course towards a more malignant behavior. Thus, our findings have supported Lin's results and provided an important insight into the role of miR-302 in tumorigenesis.

Despite a significant difference between miR- 302b expression in tumor and non-tumor samples, the data obtained from ROC analysis suggested that miR-302b has a low sensitivity and specificity in discriminating between tumor and non-tumor gastric samples. The fact that the expression level of miR-302b in both tumor and non-tumor gastric samples was low (CT=35.5 ± 2) has suggested that it is not suitable to be used as a reliable tumor marker for detection and classification of gastric cancers. However, more work is needed on the expression and function of miR-302 on different tumor types before we can assign a general role for miR-302 in tumor initiation and progression.

## Conclusion

Our data revealed a down-regulation of miR- 302b in gastric tumor samples. However, the expression demonstrated to have a low sensitivity and specificity to discriminate between the tumor and non-tumor state of the samples. The data suggest a potential tumor-suppressor role for miR-302b in tumorigenesis of gastric tissue.
